# Impact of the COVID-19 pandemic on the manifestation and course of mental illness in elderly

**DOI:** 10.1192/j.eurpsy.2024.684

**Published:** 2024-08-27

**Authors:** V. Pochueva, T. Safarova, O. Yakovleva, V. Sheshenin

**Affiliations:** ^1^FSBSI “Mental Health Research Center”, Moscow, Russian Federation

## Abstract

**Introduction:**

COVID-19 is a multisystem disease affecting not only the respiratory, gastroenterstitial and vascular systems, but also the central nervous system, which leads to a wide range of neurological and mental complications. 3 years of experience in combating the pandemic has shown that elderly people burdened with chronic somatic diseases are the most vulnerable risk group for the development of severe course and complications of COVID-19.

**Objectives:**

To study the impact of COVID-19 on the onset and course of mental illness in elderly inpatients

**Methods:**

We examined 67 inpatients aged 50 to 95 years with various mental pathologies, who underwent COVID-19 from February 2020 to December 2021. 46 people had previous history of mentall disease (PHMD), in 21 cases the disease developed for the first time. Statistical analysis was performed.

**Results:**

In the manifest group of patients, depressive episodes predominated (42.9%), including psychotic episodes (9.5%). In 28.6% of cases, organic disorders were diagnosed in the form of emotional lability, organic depression, mild cognitive impairment and delirium. In 23.8% of patients, neurotic disorders were observed in the form of depressive reactions, panic and generalized anxiety disorder. In one case (4.8%), acute polymorphic psychosis with symptoms of schizophrenia was diagnosed. The PHMD group includes affective disorders - 45.7%; organic disorders, including dementia 26.1%; schizophrenic spectrum disorders -19.6% and neurotic somatoform disorders - 8.7%. In the acute and subacute periods of COVID-19, acute psychotic states (APS) developed in both groups of patients (in 23.3% and 30.4%, respectively) in the form of delirium, psychotic depression, or polymorphic psychosis. APS were more common in PHMD patients with organic (50%) and schizophrenic spectrum disorders (33.3%) with a predominance of delirium. In the long-term period of COVID-19, PHMD patients more often than non-PHMD (60.9% and 38.1%) developed cognitive impairment (CI), especially in schizophrenia-like (77.8%) and organic (83.3%) disorders. CI developed twice as often after APS (89.5% and 39.6%, p<0.001), reaching the degree of dementia in 15.8% of cases. APS were significantly associated (p<0.05) with the development of CI (0.567733), the age of patients (0.410696) and the presence of previous cerebrovascular insufficiency (0.404916).

**Conclusions:**

The age-related features of the mental consequences of COVID-19 are the occurrence of APS in the acute period of infection and the deterioration of cognitive activity at a remote stage. The PHMD patients, especially with disorders of organic and schizophrenic spectrum, were found to be more vulnerable to the effects of COVID-19. In them, the occurrence of APS was a risk factor for the development of dementia, while in primary diseased, and patients with affective and neurotic disorders, CI was reversible or had the character of a mild cognitive disorder.

**Disclosure of Interest:**

None Declared

